# Bax-Induced Apoptosis in Leber's Congenital Amaurosis: A Dual Role in Rod and Cone Degeneration

**DOI:** 10.1371/journal.pone.0006616

**Published:** 2009-08-12

**Authors:** Séverine Hamann, Daniel F. Schorderet, Sandra Cottet

**Affiliations:** 1 IRO, Institute for Research in Ophthalmology, Sion, Switzerland; 2 Department of Ophthalmology, University of Lausanne, Lausanne, Switzerland; 3 School of Life Sciences, Federal Institute of Technology (EPFL), Lausanne, Switzerland; University of Hong Kong, Hong Kong

## Abstract

Pathogenesis in the *Rpe65^−/−^* mouse model of Leber's congenital amaurosis (LCA) is characterized by a slow and progressive degeneration of the rod photoreceptors. On the opposite, cones degenerate rapidly at early ages. Retinal degeneration in *Rpe65* ^−/−^ mice, showing a null mutation in the gene encoding the retinal pigment epithelium 65-kDa protein (Rpe65), was previously reported to depend on continuous activation of a residual transduction cascade by unliganded opsin. However, the mechanisms of apoptotic signals triggered by abnormal phototransduction remain elusive. We previously reported that activation of a Bcl-2-dependent pathway was associated with apoptosis of rod photoreceptors in *Rpe65^−/−^* mice during the course of the disease. In this study we first assessed whether activation of Bcl-2-mediated apoptotic pathway was dependent on constitutive activation of the visual cascade through opsin apoprotein. We then challenged the direct role of pro-apoptotic Bax protein in triggering apoptosis of rod and cone photoreceptors.

Quantitative PCR analysis showed that increased expression of pro-apoptotic Bax and decreased level of anti-apoptotic Bcl-2 were restored in *Rpe65^−/−^/Gnat1^−/−^* mice lacking the *Gnat1* gene encoding rod transducin. Moreover, photoreceptor apoptosis was prevented as assessed by TUNEL assay. These data indicate that abnormal activity of opsin apoprotein induces retinal cell apoptosis through the Bcl-2-mediated pathway. Following immunohistological and real-time PCR analyses, we further observed that decreased expression of rod genes in *Rpe65*-deficient mice was rescued in *Rpe65^−/−^/Bax^−/−^* mice. Histological and TUNEL studies confirmed that rod cell demise and apoptosis in diseased *Rpe65^−/−^* mice were dependent on Bax-induced pathway. Surprisingly, early loss of cones was not prevented in *Rpe65^−/−^/Bax^−/−^* mice, indicating that pro-apoptotic Bax was not involved in the pathogenesis of cone cell death in *Rpe65*-deficient mice.

This is the first report, to our knowledge, that a single genetic mutation can trigger two independent apoptotic pathways in rod and cone photoreceptors in *Rpe65*-dependent LCA disease. These results highlight the necessity to investigate and understand the specific death signaling pathways committed in rods and cones to develop effective therapeutic approaches to treat RP diseases.

## Introduction

Mutations in the gene encoding the protein RPE65 are associated with several forms of inherited retinal dystrophies, such as autosomal recessive retinitis pigmentosa (RP) [1] and autosomal recessive childhood-onset severe retinal dystrophy [2] which are characterized by profound visual deficiency, night blindness and reduced or non detectable electroretinogram (ERG). LCA consists in a severe form of early-onset autosomal recessive RP and is due in 6 to 15% of the cases to mutations in the *Rpe65* gene [3]. RPE65, abundantly expressed in the RPE, is the retinoid isomerase responsible to generate 11-*cis*-retinal necessary for light-induced phototransduction mediated by the visual pigments rod and cone opsins [4]–[9]. In the absence of the RPE65 protein, rod opsin apoprotein triggers light-independent, constitutive activation of the visual cascade resulting in photoreceptor cell loss [10]. Human RPE65-LCA is characterized as a rod-cone dystrophy with severe retinal defect in the first decades of life. The clinical outcome of this disease has mainly been attributed to rod and cone loss, although the molecular pathways leading to retinal cell death still remain to be identified [11], [12].

*Rpe65*-deficient mice are a murine model of LCA. These mice exhibit changes in retinal morphology, function, and biochemistry resembling the alterations seen in human LCA patients, including severely depressed light- and dark-adapted ERG responses [4], [13]. In the absence of RPE65, rod photoreceptors display a slow and progressive degeneration [4] dependent on continuous activation of residual transduction cascade by unliganded opsin [10]. Of note is also the early disorganization and loss of photoreceptor outer segments (OS) [4]. The remaining visual capacity is attributed to small amount of 9-*cis*-retinal forming the photosensitive isorhodopsin in the rods [14]. The residual ERG recording, first evaluated as cone response, actually corresponded to rod function mimicking cone function by responding under normally cone-isolating lighting conditions [9]. Recent studies showed that the decreased expression of cone-specific opsins and transducin correlated with cone degeneration at early ages in *Rpe65*-deficient retinas [15]–[17]. These data indicate that Rpe65 deficiency affects more severely cones than rods in mice.

Several studies highlighted that many of the molecular pathways involved in ocular diseases rely on mitochondria-dependent apoptotic pathway involving proteins of the Bcl-2 family [18]. Bcl-2 members, known as key regulatory proteins in apoptotic events, can promote either cell survival or cell death. Indeed, the relative amount or equilibrium between pro- and anti-apoptotic members is crucial to sensitize cell fate towards survival or apoptosis. The anti-apoptotic action of Bcl-2 acts through binding and inhibiting pro-apoptotic effector proteins Bax and Bak. The latter promote apoptosis by altering mitochondrial functions and activating the release of downstream apoptogenic factors [19]. Anti- and pro-apoptotic Bcl-2 members are thought to play a role in the pathogenesis of several retinal disorders. While expression of Bax is upregulated following ischemia-induced retinal injury in rat [20], concomitant decreased Bcl-2 and increased Bax protein levels have been reported after elevated intraocular pressure in murine glaucoma model [21]. Following experimental retinal detachment, photoreceptor cell death is abolished in Bax^−/−^ mice, suggesting a critical role for Bax-mediated apoptosis [22]. Similarly, retinas of double knock-out Bax^−/−^/Bak^−/−^ mice have been shown to be resistant to light-induced photoreceptor degeneration [23]. On the opposite, Bax deficiency is described to be insufficient to protect photoreceptors from death in *rd* mouse, a fast degenerative RP model characterized by a homozygous mutation in the *Pde6b* gene encoding rod-specific cGMP phosphodiesterase [24].

We previously observed that anti- and pro-apoptotic genes of the Bcl-2 family were differentially regulated during the development of LCA in the *Rpe65^−/−^* mouse model [25]. Moreover, we reported that activation and translocation of pro-apoptotic Bax to mitochondria was associated with apoptosis of rod photoreceptors as the disease progressed [26]. In this study, we first assessed whether triggering of Bcl-2-related apoptotic pathway was mediated by constitutive phototransduction signaling. We further challenged whether disruption of pro-apoptotic Bax was sufficient to prevent rod and cone photoreceptor cell death.

## Results

### Phototransduction-dependent Apoptosis of Photoreceptors Is Mediated by Activation of the Bcl-2 Apoptotic Pathway In *Rpe65*-deficient Mice

Our previous work [25], [26], showing that activation of the Bcl-2-related signaling pathway was associated with retinal degeneration in *Rpe65*-deficient mice, led us to investigate whether this apoptotic pathway was triggered by light-independent, constitutive activity of the phototransduction cascade.

**Figure 1:**
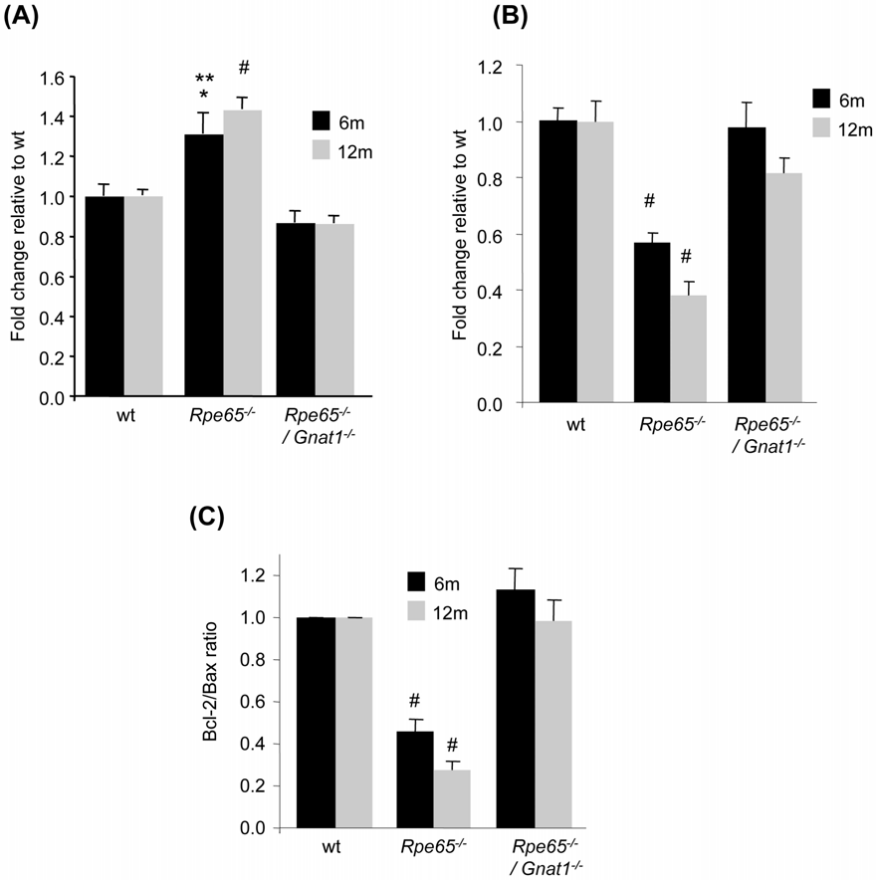
Constitutive Opsin Apoprotein Signaling Activity Triggers Bcl-2-related Apoptotic Pathway in *Rpe65*-deficient Mice. Real-time PCR analysis in 6 month-old (6 m, black square) and 12 month-old (12 m, grey square) wt, *Rpe65^−/−^* and *Rpe65^−/−^/Gnat1^−/−^* retinas showing that (A) increased transcriptional expression of pro-apoptotic Bax and (B) reduced level of anti-apoptotic Bcl-2 mRNA observed in *Rpe65*-deficient retinas were rescued in *Rpe65^−/−^/Gnat1^−/−^* retinas lacking functional rod transducin. (C) Restoration of impaired equilibrium between normalized levels of Bcl-2 toward Bax members of the Bcl-2 family of proteins, expressed as Bcl-2/Bax ratio. Data are the mean±SE of six independent experiments. * p<0.05 by ANOVA test for *Rpe65^−/−^ versus* wt ; ** p<0.001 by ANOVA test for *Rpe65^−/−^ versus Rpe65^−/−^/Gnat1^−/−^*; # p<0.001 by ANOVA test for *Rpe65^−/−^ versus* wt and *Rpe65^−/−^/Gnat1^−/−^*.

Regulation of Bcl-2 and Bax gene expression was assessed by real-time PCR in retinas of wt, *Rpe65^−/−^* and *Rpe65^−/−^/Gnat1^−/−^* mice at 6 and 12 months of age. As depicted in figure 1A, whereas Bax expression was increased by 1.5-fold in 6 and 12 month-old *Rpe65*-deficient mice, transcriptional upregulation of the pro-apoptotic gene was abolished in *Rpe65^−/−^/Gnat1^−/−^* mice in which phototransduction signaling was blocked in the absence of functional rod transducin. Similarly, the strong decrease in Bcl-2 mRNA in *Rpe65^−/−^* retinas as the disease progresses, as reflected by a 40% reduction at 6 months and a 60% reduction at 12 months, was prevented in *Rpe65^−/−^/Gnat1^−/−^* retinas (Figure 1B). This resulted in rescue of the impaired balance between anti- and pro-apoptotic expressed genes observed in *Rpe65*-deficient mice, with Bcl-2/Bax ratio of 0.46±0.06 and 0.28±0.05 at 6 and 12 months, respectively (Figure 1C). This is in close correlation with our previous observation showing decreased ratio of Bcl-2 toward Bax at the protein level in *Rpe65^−/−^* mice during the course of the disease [26].

**Figure 2:**
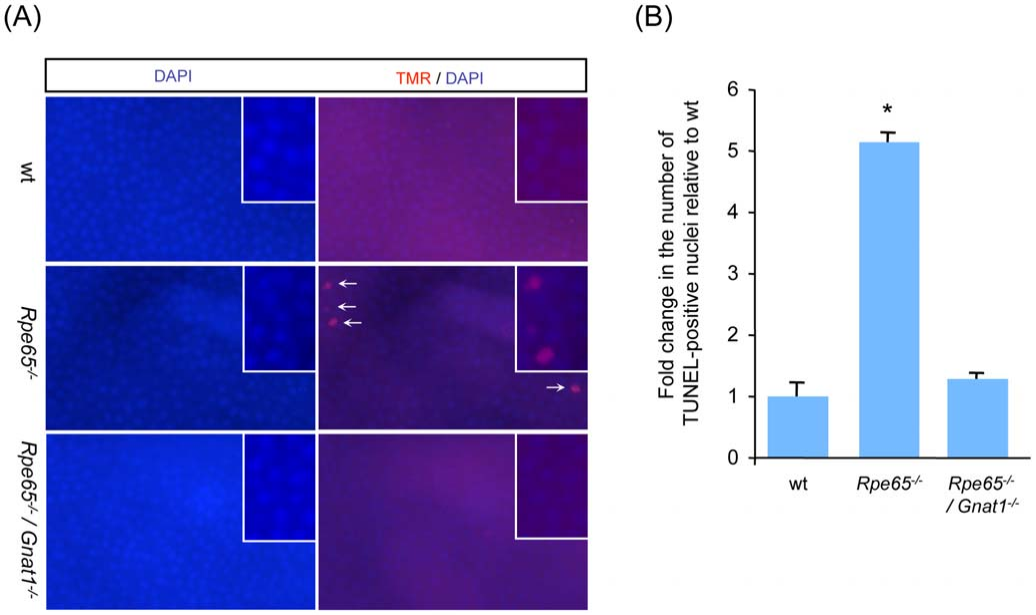
Bcl-2-related Apoptosis of Photoreceptors Is Prevented in Phototransduction-deficient *Rpe65^−/−^/Gnat1^−/−^* Mice. (A) TUNEL assay was performed in 6 month-old wt, *Rpe65^−/−^* and *Rpe65^−/−^/Gnat1^−/−^* retina flatmounts (400×) labeled with TMR nucleotides to detect red fluorescence staining of apoptotic photoreceptor nuclei. Counterstaining with DAPI was performed to localize faced up photoreceptors. (B) Increase in the number of apoptotic cells relative to wt retinas was measured by counting TUNEL-positive nuclei. Double knock-out mice deficient for rod transducin were protected from phototransduction-induced apoptosis as compared in *Rpe65*-deficient mice. Data are the mean±SE of three independent experiments. * p<0.001 by ANOVA test for *Rpe65^−/−^ versus* wt and *Rpe65^−/−^/Gnat1^−/−^*

To address whether inhibition of Bcl-2-related apoptotic pathway was sufficient to prevent apoptosis of photoreceptors in diseased retinas, retinal cell apoptosis was investigated by TUNEL assay in retina flatmounts in 6 month-old wt, *Rpe65^−/−^* and *Rpe65^−/−^/Gnat1^−/−^* mice (Figure 2A). When counting fluorescent-labelled TUNEL-positive nuclei, a 5-fold increase in the number of apoptotic photoreceptors was observed in *Rpe65^−/−^* retinas as compared with wt retinas (5.14±0.14 in *Rpe65^−/−^* vs 1.0±0.22 in wt), whereas cell death was prevented in *Rpe65^−/−^/Gnat1^−/−^* retinas (1.29±0.08) (Figure 2B).

Altogether, these results demonstrate that abnormal, unliganded opsin signaling activity induces photoreceptor apoptosis in *Rpe65*-mediated LCA disease triggered by Bcl-2-related apoptotic pathway.

### Disruption of Bax in *Rpe65*-deficient Mice

To assess the role of Bax as a main pro-death effector of photoreceptor apoptosis in the *Rpe65^−/−^* mouse model of LCA, we generated *Rpe65^−/−^/Bax^−/−^* mutant mice both deficient for *Rpe65* and *Bax*.

Analysis of genomic DNA in multiplex PCR reaction using specific primers hybridizing to wild-type and mutant alleles allowed to assess the genotype of the wt, *Rpe65^−/−^*, *Bax^−/−^* and *Rpe65^−/−^/Bax^−/−^* mouse strains (Figure S1A). To confirm that Bax transcript was disrupted in *Bax^−/−^* mice, RT-PCR was performed on total RNA isolated from mouse retinas. No Bax-specific RT-PCR product was seen in either *Bax^−/−^* or *Rpe65^−/−^/Bax^−/−^* mice as compared with wt and *Rpe65^−/−^* mice, whereas control Gapdh transcript was amplified in all retina samples (Figure S1B).

**Figure 3:**
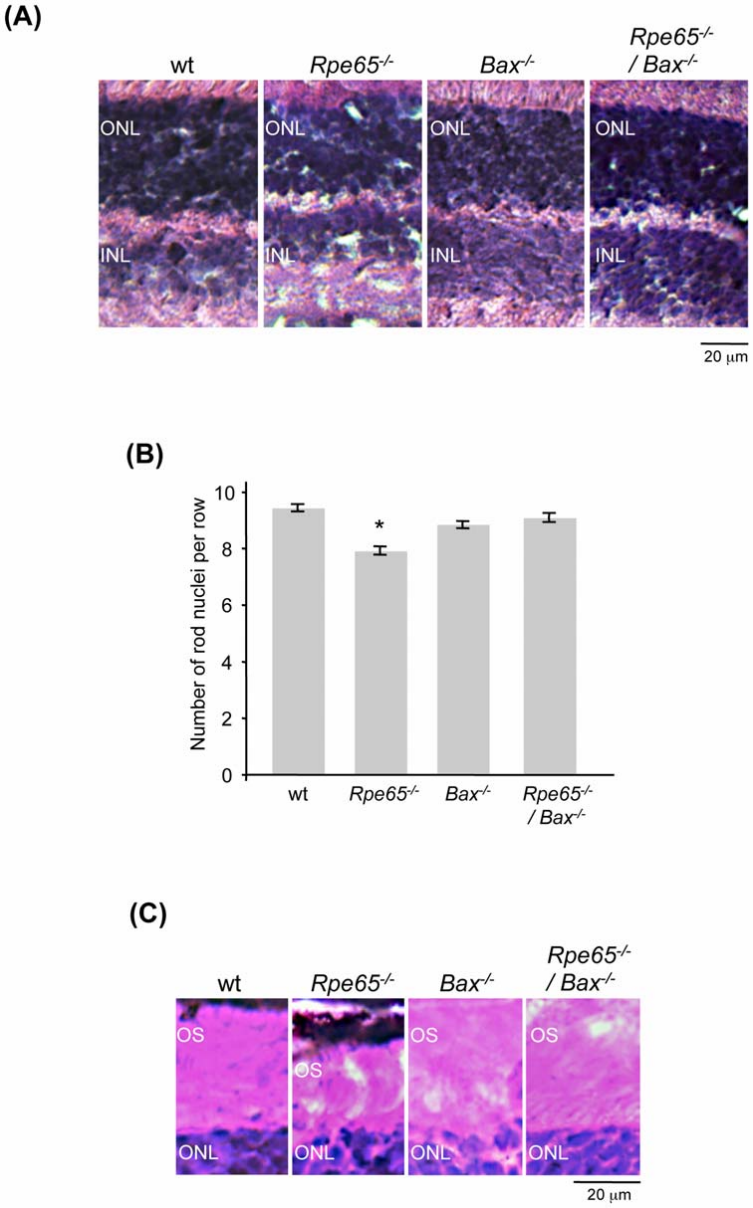
Histological Analysis of *Rpe65^−/−^/Bax^−/−^* Retinas at 2 Months of Age. (A) Examination of retinal morphology (600×) and (B) Counting rows of rod nuclei within the ONL of *Bax*-deficient retinas showed no increase in outer nuclear layer thickness as compared with wt retinas. A slight decrease in ONL thickness was only shown in *Rpe65^−/−^* retinas. * p<0.001 by ANOVA test for *Rpe65^−/−^ versus* wt, *Bax^−/−^* and *Rpe65^−/−^/Bax^−/−^*. (C) Early shortening of OS length at the onset of retinal degeneration in *Rpe65^−/−^* mice was not observed in *Rpe65^−/−^/Bax^−/−^* mice. ONL, outer nuclear layer; INL, inner nuclear layer; OS, outer segment.

We performed histological analysis at 2 months of age to examine the retinal morphology of *Bax^−/−^* mice on a *Rpe65*-null genetic background. As shown in previous studies, we confirmed following morphological examination (Figure 3A) and counting of rod nuclei (Figure 3B) that no change in outer nuclear layer (ONL) thickness, containing the photoreceptor nuclei, was obvious in *Bax*-deficient retinas as compared to wt retinas. A slight decrease in ONL thickness was only observed in *Rpe65^−/−^* mice. It was previously reported that the increased number of cells in the inner nuclear layer (INL) and ganglion cell layer (GCL) by adulthood in *Bax*-deficient mice correlated with a corresponding decrease in physiological apoptosis during retinal development and not to ongoing proliferation as assessed by BrdU incorporation [22], [24], [27]–[28]. We also observed that the secondary neuronal layers were increased in *Bax*-deficient retinas, further confirming the genotype of *Rpe65^−/−^/Bax^−/−^* mice (Figure 3A). Disorganization and shortening of outer segments are early markers of retinal degeneration in *Rpe65* ^−/−^ retinas [4]. While the length of OS was slightly decreased in *Rpe65^−/−^* retinas, this shortening was not observed in *Rpe65*-deficient retinas lacking Bax, suggesting that early in the disease OS morphology is preserved in the absence of Bax (Figure 3C).

### Rod Photoreceptors Are Protected from Apoptosis in *Rpe65*-deficient LCA Mice Lacking Pro-apoptotic Bax

**Figure 4:**
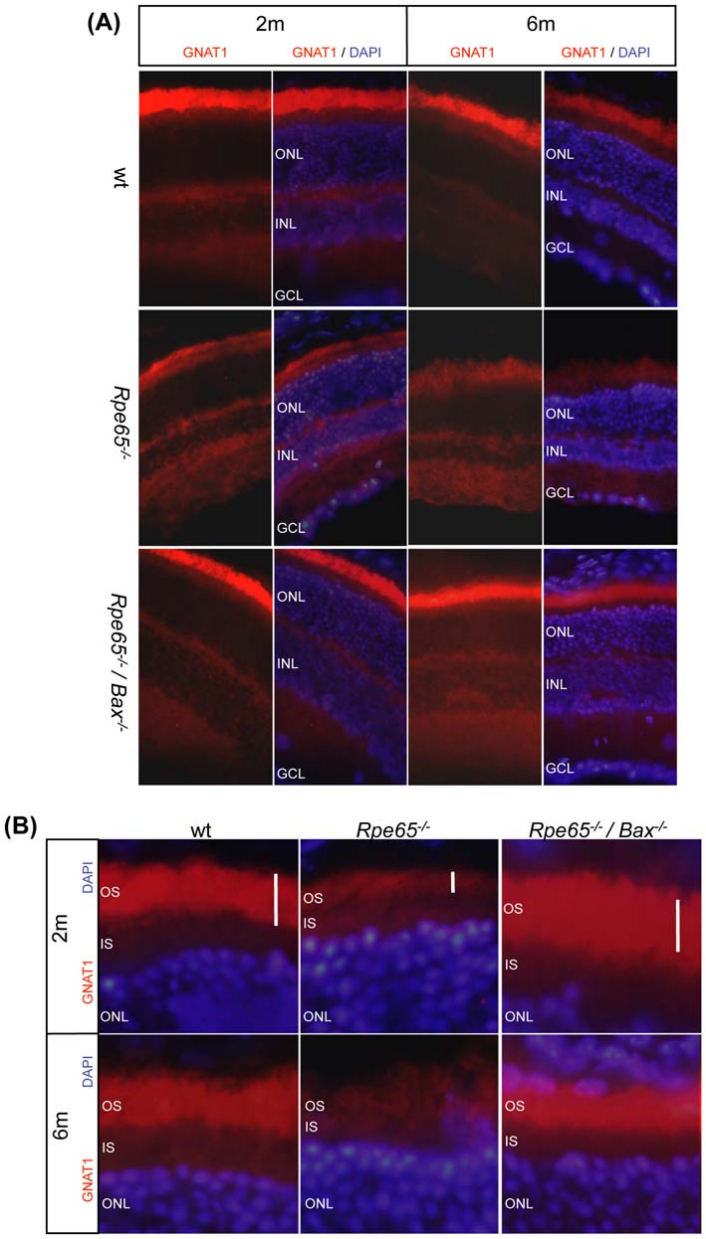
Immunohistological Analysis of Rod Transducin Expression during the Course of the Disease. (A) Immunohistological staining (600×) with gnat1 antibody (*GNAT1 panels*) showing expression of transducin in rod OS at 2 (2 m) and 6 (6 m) months of age. Counterstaining with DAPI was performed to identify the retinal cell layers (*GNAT1/DAPI panels*). While the amount of transducin was decreased in *Rpe65^−/−^* retinas at both ages, as compared with wt retinas, expression of the protein was restored in *Rpe65^−/−^/Bax^−/−^* retinas. (B) Higher magnification of immunostained retina sections showing that OS shortening in *Rpe65*-deficient retinas was fully preserved at both ages in *Rpe65^−/−^* retinas lacking Bax. ONL, outer nuclear layer; INL, inner nuclear layer; GCL, ganglion cell layer; IS, inner segment; OS, outer segment.

**Figure 5:**
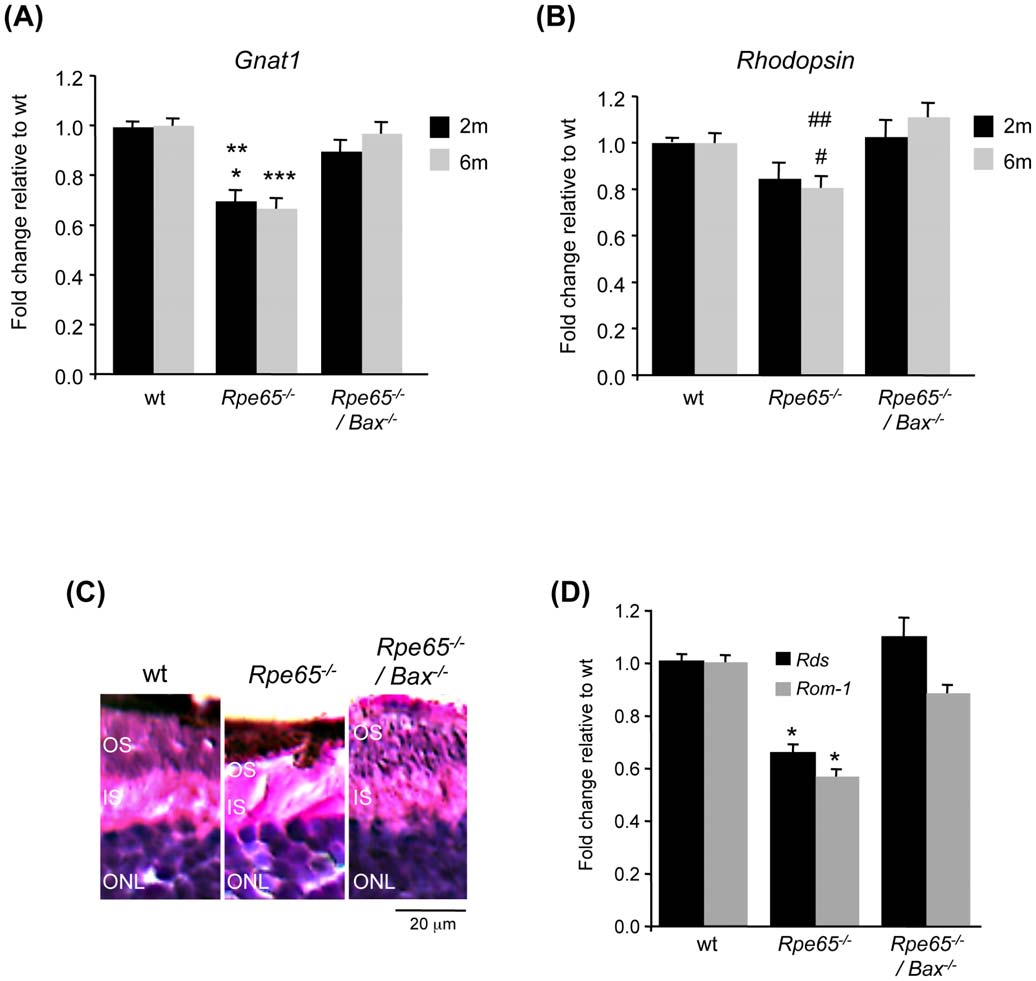
Decreased Expression of Rod Photoreceptor Markers Was Prevented in *Rpe65^−/−^/Bax^−/−^* Mice. (A) rod transducin and (B) rhodopsin mRNA levels were assessed by quantitative PCR in 2 month-old (2 m, black square) and 6 month-old (6 m, grey square) wt, *Rpe65^−/−^* and *Rpe65^−/−^/Bax^−/−^* mice. Decreased expression of rod-specific transcripts in *Rpe65*-deficient retinas was restored in *Rpe65^−/−^/Bax^−/−^* retinas at both ages. Data are the mean±SE of three (6 m) to four (2 m) independent experiments. * p<0.001 by ANOVA test for *Rpe65^−/−^ versus* wt; ** p<0.01 by ANOVA test for *Rpe65^−/−^ versus Rpe65^−/−^/Bax^−/−^*; *** p<0.001 by ANOVA test for *Rpe65^−/−^ versus* wt and *Rpe65^−/−^/Bax^−/−^*; # p<0.05 by ANOVA test for *Rpe65^−/−^ versus* wt; ## p<0.001 by ANOVA test for *Rpe65^−/−^ versus Rpe65^−/−^/Bax^−/−^*. (C) Histological analysis (600×) showing almost complete disrupted rod OS in *Rpe65^−/−^* retinas at 6 months, as compared to preserved OS structure in wt and *Rpe65^−/−^/Bax^−/−^* retinas. (D) Quantitative PCR analysis of expressed *Rds* and *Rom-1* OS marker genes in 6 month-old mice. Data are the mean±SE of three independent experiments. * p<0.001 by ANOVA test for *Rpe65^−/−^ versus* wt and *Rpe65^−/−^/Bax^−/−^*. ONL, outer nuclear layer; IS, inner segment; OS, outer segment.

To further investigate the role of Bax in retinal degeneration, we performed immunohistological analysis of rod transducin (*Gnat1*) expression in *Rpe65^−^* ^/−^ mice during the course of the disease at 2 and 6 months of age. Counterstaining with DAPI was performed to identify the retinal cell layers. Immunohistological staining with gnat1 antibody showed localized expression of transducin in rod OS (Figure 4). Decreased expression of rod transducin (Figure 4A) observed in *Rpe65^−^* ^/−^ mice at both ages was rescued in *Rpe65^−/−^/Bax^−/−^* mice, as compared with wt animals. Restored levels of rod-specific markers transducin (Figure 5A) and rhodopsin (Figure 5B) were further confirmed by real-time PCR analysis. Furthermore, impaired OS structure in *Rpe65^−/−^* mice, already obvious by 2 months of age (Figures 3C and 4B), was highly disrupted and reduced as the disease progresses by 6 months of age, as shown in immunohistological (Figure 4B) and histological (Figure 5C) analyses. This was correlated with decreased expression of the OS markers *Rds* and *Rom-1* (Figure 5D). However, OS morphological defects as well as decreased mRNA levels of the OS-specific structural proteins were rescued in diseased retinas lacking pro-apoptotic Bax.

**Figure 6:**
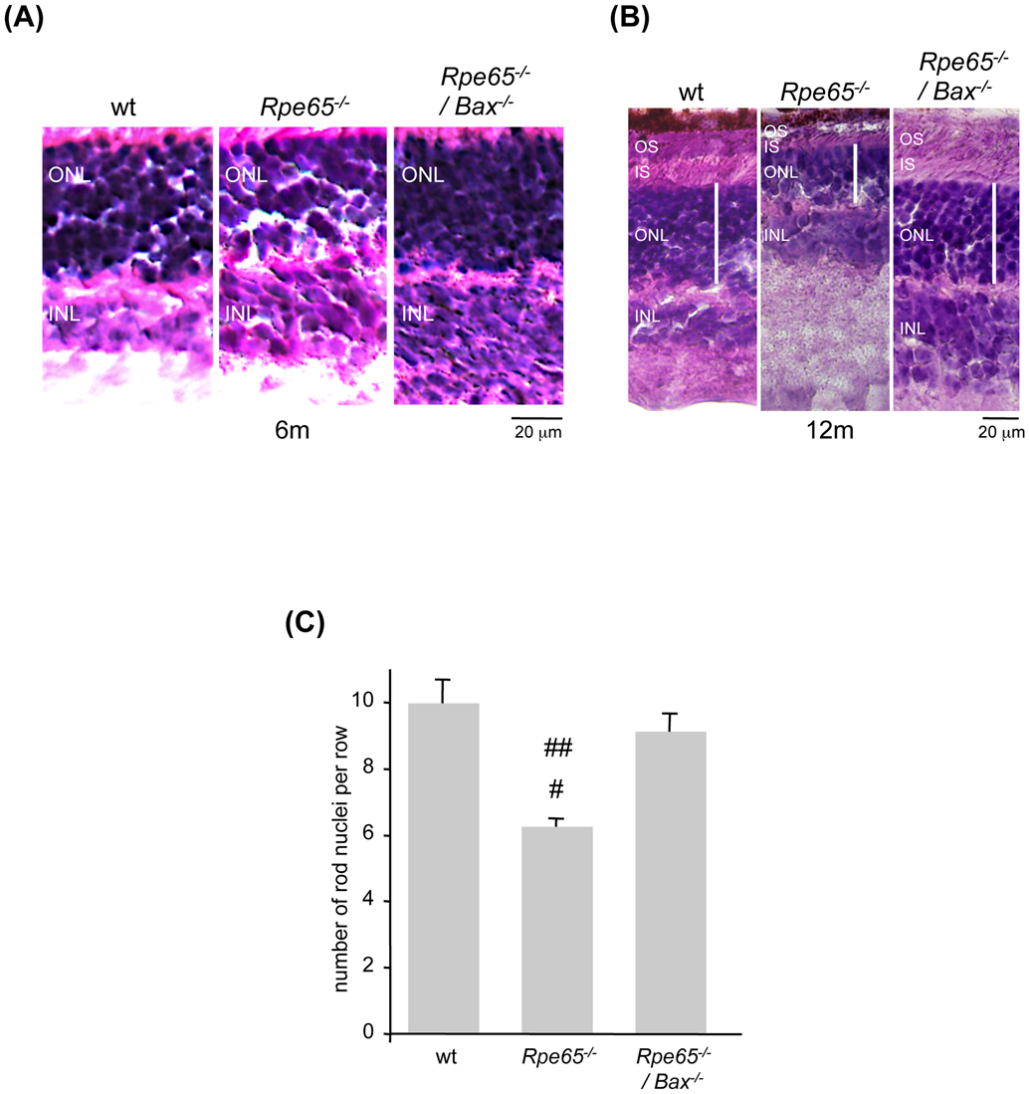
Disruption of Pro-apoptotic Bax Was Sufficient to Prevent Rod Photoreceptor Defect and Loss in *Rpe65*-deficient Animals. (A) Examination of retinal morphology (600×) and (C) counting rows of rod nuclei at 6 months (6 m) of age showed that whereas ONL thickness was reduced in *Rpe65^−/−^* retinas, loss of photoreceptor nuclei was prevented in *Rpe65*-deficient retinas lacking Bax. The data presented are the mean±SE of four independent experiments. # p<0.01 by ANOVA test for *Rpe65^−/−^ versus* wt; ## p<0.05 by ANOVA test for *Rpe65^−/−^ versus Rpe65^−/−^/Bax^−/−^*. (B) Histological analysis at 12 months (12 m) of age showing long-term protection of ONL and OS integrity in the absence of pro-apoptotic Bax in *Rpe65^−/−^/Bax^−/−^* mice. ONL, outer nuclear layer; INL, inner nuclear layer; IS, inner segment; OS, outer segment.

**Figure 7:**
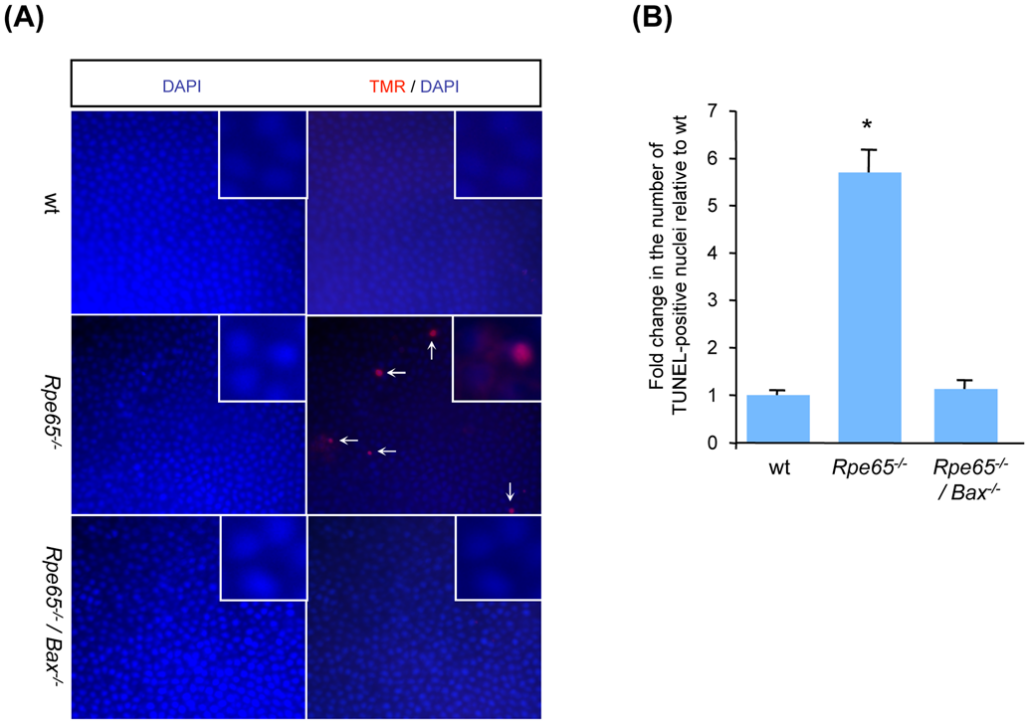
Bax-dependent Rod Photoreceptor Apoptosis Was Abolished in *Rpe65^−/−^/Bax^−/−^* Mice. (A) TUNEL assay was performed in 6 month-old wt, *Rpe65^−/−^* and *Rpe65^−/−^/Bax^−/−^* retina flatmounts (400×) labeled with TMR nucleotides to detect red fluorescence staining of apoptotic rod nuclei. Counterstaining with DAPI was performed to localize faced up photoreceptor cells. (B) Increase in the number of apoptotic TUNEL-positive cells in *Rpe65^−/−^* retinas, relative to wt retinas, was abolished in *Rpe65^−/−^/Bax^−/−^* retinas. Data are the mean±SE of three independent experiments. * p<0.001 by ANOVA test for *Rpe65^−/−^ versus* wt and *Rpe65^−/−^/Bax^−/−^*.

To more accurately assess whether pro-apoptotic Bax is sufficient to trigger retinal cell death, loss of rod photoreceptor nuclei was investigated. Retinal degeneration was assessed by histological analysis (Figure 6A) and counting rows of the surviving photoreceptor nuclei in the ONL at 6 months of age (Figure 6C). The number of rod nuclei was reduced by about 30 to 40% in *Rpe65^−/−^* retinas, which is in accordance with previous observations [4], [10], [29], whereas ONL thickness was preserved in double knock-out retinas as compared with wt retinas. More importantly, histological analysis at 12 months of age not only showed a delayed retinal defect but also an efficient, long term protection of ONL and OS integrity in the absence of functional Bax (Figure 6B). To confirm that photoreceptor apoptosis was prevented in diseased retinas lacking pro-apoptotic Bax, TUNEL analysis was performed on 6 month-old retina flatmounts (Figure 7A). While the number of TUNEL-positive apoptotic rods was increased 6-fold in *Rpe65^−/−^* retinas (5.7±0.46) as compared with wt retinas (1.0±0.08), apoptosis of rod photoreceptors was prevented in *Rpe65^−/−^/Bax^−/−^* retinas (1.13±0.17) (Figure 7B). Furthermore, prevention of photoreceptor cell death in *Bax*-deficient diseased retinas may not be explained by re-expression of Bcl-2 since transcriptional expression of the anti-apoptotic member was not restored (Figure S2).

Altogether, these results indicate that rod photoreceptor degeneration is triggered by a Bax-induced apoptotic pathway in *Rpe65*-dependent LCA disease.

### Early Degeneration of Cone Photoreceptors in *Rpe65*-deficient Mice Did Not Rely on Activation of Bax-mediated Apoptotic Pathway

In the *Rpe65^−/−^* mouse model of LCA, cone photoreceptors degenerate much more rapidly than rods [9], [15]–[16]. We thus addressed the question whether the early apoptotic events involved in cone cell death were also dependent on activation of pro-apoptotic Bax.

**Figure 8:**
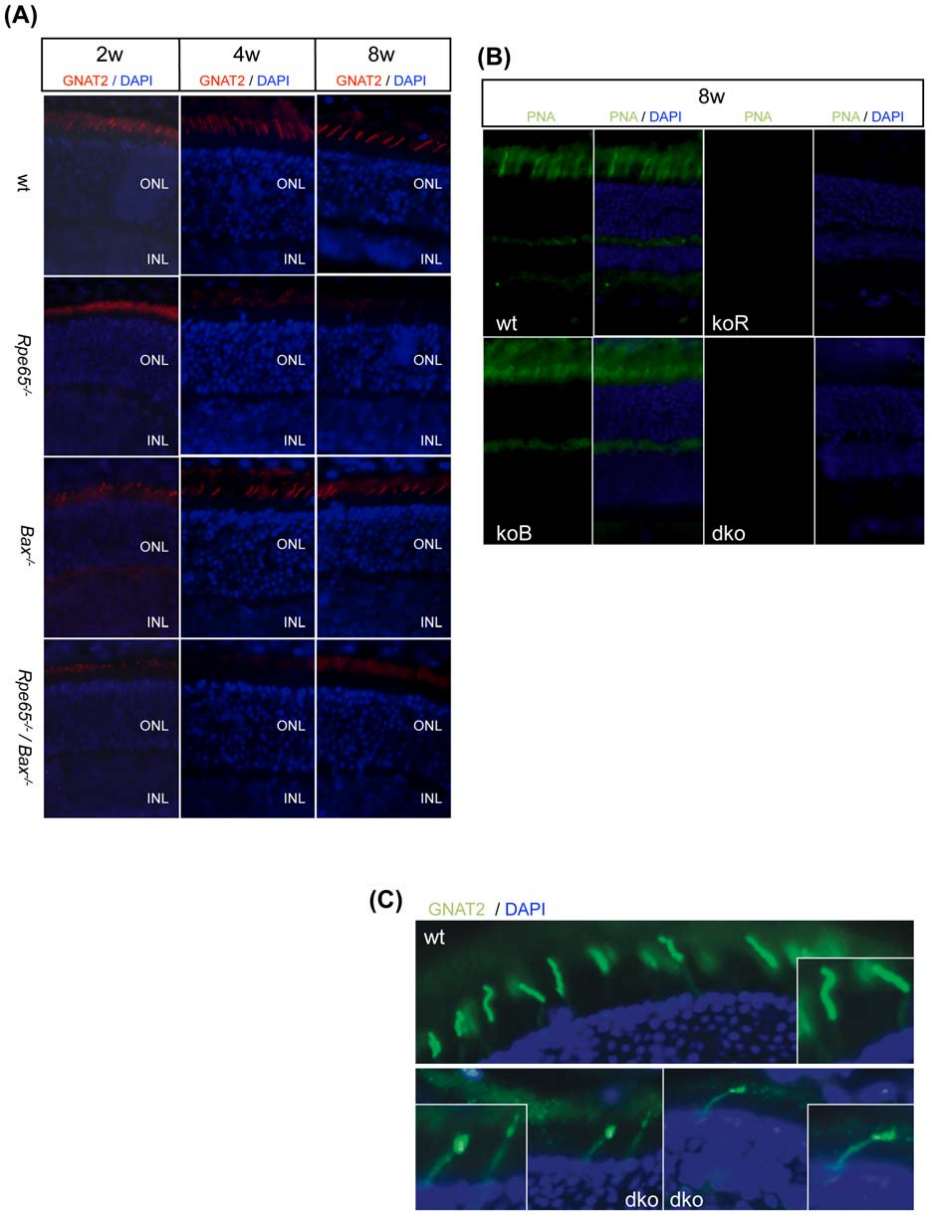
Early Degeneration of Cone Photoreceptors Was Not Prevented in *Rpe65^−/−^/Bax^−/−^* Mice. (A) Immunohistological staining (600×) with gnat2 antibody showing that early lost expression of cone transducin, as early as 2 weeks of age, was not prevented in *Rpe65^−/−^/Bax^−/−^* retinas as compared to *Rpe65^−/−^* retinas (2–8 w, 2–8 weeks; ONL, outer nuclear layer; INL, inner nuclear layer). (B) FITC-labelled PNA staining (600×) in 8 week-old (8 w) *Rpe65*-deficient retinas confirmed that degenerating cones were not rescued in the absence of Bax. (C) Immunohistological analysis demonstrating that cone transducin failed to properly traffic to the OS in the few surviving cones still present at the retinal periphery at 6 months of age in *Rpe65^−/−^/Bax^−/−^* mice as compared to wt mice. Counterstaining with DAPI allowed for the identification of photoreceptor cell nuclei. koR, *Rpe65^−/−^*; koB, *Bax^−/−^*; dko, *Rpe65^−/−^/Bax^−/−^*.

**Figure 9:**
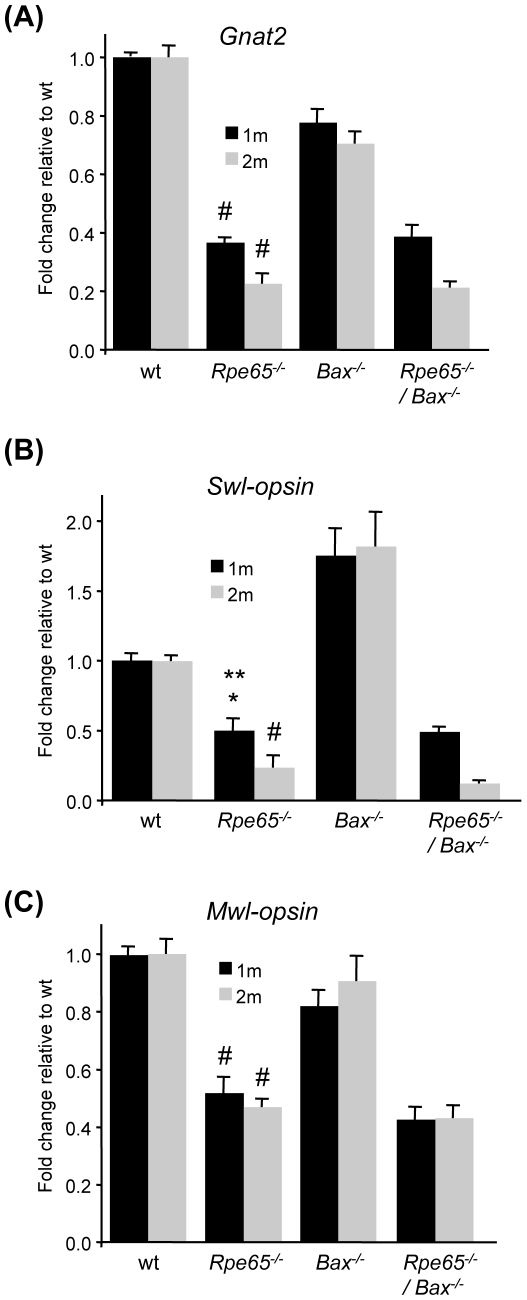
Loss of Cones in *Rpe65^−/−^/Bax^−/−^* Mice Was Confirmed by qPCR Analysis of Cone-specific Markers. Strong decrease in transcriptional expression of (A) Gnat2, (B) Swl-opsin and (C) Mwl-opsin at 1 (1 m) and 2 months (2 m) of age in *Rpe65^−/−^* and *Rpe65^−/−^/Bax^−/−^* retinas indicated that loss of cones was not rescued in the absence of pro-apoptotic Bax. Data are the mean±SE of four independent experiments. # p<0.001 by ANOVA test for *Rpe65^−/−^ versus* wt and *Bax^−/−^*; * p<0.05 by ANOVA test for *Rpe65^−/−^ versus* wt; ** p<0.001 by ANOVA test for *Rpe65^−/−^ versus Bax^−/−^*.

By immunostaining analysis, early and fast reduction in cone transducin (*Gnat2*) expression was observed between 2 and 8 weeks of age in *Rpe65^−/−^* retinas as compared to wt and *Bax^−/−^* retinas (Figure 8A). However, in contrary to our observation in rod photoreceptors, decreased level of cone transducin was not restored in *Rpe65^−/−^/Bax^−/−^* mice lacking pro-apoptotic *Bax* (Figure 8A). Specific staining of cone extracellular matrix with FITC-labelled PNA further showed that, by 2 months of age, cones were lost both in *Rpe65^−/−^* and *Rpe65^−/−^/Bax^−/−^* retinas (Figure 8B). Moreover, cone transducin failed to properly traffic to the OS in the few surviving cones at later times in peripheral retina in *Rpe65^−/−^/Bax^−/−^* mice (Figure 8C). This observation correlates with previous reports describing impaired localization of cone opsins in synaptic pedicle and IS in residual cones almost exclusively found at the periphery of *Rpe65^−/−^* retinas [16], [17]. Confirmation of the loss of cone-specific markers in *Rpe65^−/−^/Bax^−/−^* mice, similarly to what was observed in *Rpe65^−/−^* mice, was demonstrated by quantitative PCR study of cone transducin (Gnat2) and cone opsins (short wavelength (Swl) and middle wavelength (Mwl)-opsins) transcriptional expression at 1 and 2 months of age (Figure 9). Decreased expression of Gnat2 and Swl-opsin was more pronounced at 2 months of age in *Rpe65*-deficient retinas as well as in *Rpe65^−/−^/Bax^−/−^* retinas.

Altogether, these results demonstrate that Bax-induced signaling pathway is not critical for mislocalization of the cone-specific phototransduction components and not sufficient to induce early degeneration of cones in *Rpe65*-deficient LCA mice.

## Discussion

In the current study, we reported in the *Rpe65^−/−^* mouse model of LCA that photoreceptor apoptosis induced by continuous transducin-mediated signaling is triggered by the Bcl-2-mediated apoptotic pathway. Indeed, we observed that altered regulation of pro-and anti-apoptotic members of the Bcl-2 family was prevented in *Rpe65^−/−^/Gnat1^−/−^* mice lacking functional transducin. Moreover, the restored equilibrium between Bax and Bcl-2 was associated with the survival of the photoreceptors in diseased retinas. Several lines of evidence show that mutations activating persistent visual transduction can cause congenital stationary night blindness and retinal degeneration. Deficiency in the visual cycle, during vitamin A deprivation in diet [30], [31] or in *Lrat^−/−^* mice [32], also leads to over-stimulation of the transduction cascade through unliganded opsin. As for the described mutations in RPE65, affected patients with mutations in the gene encoding the lecithin retinol acyl transferase (LRAT) develop severe early-onset retinal dystrophy [33]. Other defects in visual transduction, such as constitutively active mutant opsins [34]–[36], mutations in cyclic guanosine monophosphate-gated channel [37] or in retinal guanylate cyclase [38], [39], are associated with photoreceptor dystrophies for which continuous activation of the visual cascade is the most likely explanation for the outcome of the disease. Genetically engineered null mutations in arrestin [40] and rhodopsin kinase [41], prolonging light-activated rhodopsin signaling, also show similar physiological and cellular dysfunctions leading to retinal degeneration. Inhibition of transducin activity in these mice prevents continuous, moderate light-induced damage, indicating that rod photoreceptor apoptosis is triggered by prolonged light-activated transduction cascade in these mice [42].

The Bcl-2-related apoptotic pathway is activated and participates in the pathogenesis of several RP diseases [18] as well as in glaucoma [21], [43], retinal ischemia [20], [44], [45] and retinal detachment [22]. In this study, we further challenged the direct role of Bax in retinal cell death associated with RPE65 defect by disrupting the pro-apoptotic gene in the *Rpe65^−/−^* mice. Absence of the pro-apoptotic protein was sufficient to rescue the phenotype of the disease. Indeed, the altered OS structure and shortening as well as the reduced expression of rod-specific genes were rescued in *Rpe65^−/−^/Bax^−/−^* retinas. ONL thinning and apoptosis of rod photoreceptors were also prevented in the absence of functional Bax. More important, disruption of the pro-apoptotic protein provided a long term protection and preservation of the OS and ONL, still effective in older mice at 12 months of age. From our results, it is tempting to speculate that the proteins of the Bcl-2 family may trigger a common apoptotic pathway in a subset of inherited retinal defects resulting from excessive visual transduction. Future studies will be needed to address the impact of the Bcl-2-related pathway in other inherited mutations leading to constitutive phototransduction-dependent retinal degeneration.

The nature of cone dysfunction in *Rpe65* deficiency still remains to be elucidated and thus prompted us to assess whether, similarly to our observation in rods, Bax-induced apoptosis was the cause of early cone loss in *Rpe65*-deficient mice. Early and major cone degeneration was recently reported in LCA patients with RPE65 mutations [11], indicating that the murine disease phenotype is very similar to that of the human counterpart. We observed in this study that degeneration of the cones, reflected by the loss of cone-specific markers and the disruption of cone extracellular matrix, was not prevented in *Rpe65* ^−/−^ lacking the pro-apoptotic protein. Moreover, the few surviving cones observed at the peripheral retina in *Rpe65^−/−^/Bax^−/−^* mice showed mislocalized cone transducin. Impaired localization of cone opsins within the synaptic pedicle, cell body and inner segments, was previously reported in *Rpe65^−/−^* mice [16], [17]. This was similarly observed in *Lrat^−/−^* [17], retinal guanylate cyclase *GC1^−/−^* [46], [47] and cone cyclic nucleotide-gated channel *Gnga3^−/−^* [48] mice, suggesting that some genetic defects in proteins of the visual cascade may converge at a common degenerative pathway causing cone cell death. Treatment with exogenous 11-*cis*-retinal restored correct OS targeting of cone phototransduction components in *Rpe65*-deficient mice. This was associated with increased cone response and survival, suggesting that visual chromophore may act as a chaperone to improve sorting and correct trafficking of these proteins [16]–[17], [49]. These results indicate that cone loss may be attributed, at least partially, to impaired protein folding and deficient targeting triggering altered photoreceptor physiology and intracellular death cascade. It has been similarly observed that rod opsin mutations resulting in protein misfolding and impaired sorting may lead to retinal degeneration (reviewed in [50]). Protein misfolding elicits an adaptive endoplasmic reticulum-stress response and enhances protein degradation by the ubiquitin-proteasome system. If endoplasmic reticulum stress is maintained, proteasome-mediated protein degradation can culminate in apoptotic cell death involving caspase activation and mitochondrial signaling. In case the normal proteolytic machinery becomes saturated, protein aggregation in cytosol may activate other cytotoxic pathways including impaired cytoskeletal network–induced apoptosis as well as autophagic cell death [51]–[53]. Abnormal sorting of cone-specific proteins may further induce cell death through different molecular mechanisms involving impaired vesicular trafficking, interference with synaptic transmission and metabolic stress due to continuous degradation of mis-sorted proteins. Further studies in pure cone mouse models, the *Rpe65^−/−^/Nrl^−/−^* or *Rpe65^−/−^/Rho^−/−^* mice, will be necessary to identify the intracellular apoptotic pathways involved in the demise of the cones.

In summary, this is the first report showing that phototransduction-dependent apoptosis of rod photoreceptors in *Rpe65^−/−^* mice is triggered by the Bcl-2-related apoptotic pathway. The observations that Bax-induced apoptosis is responsible for progressive loss of rods, while early degeneration of cones is not mediated by pro-apoptotic Bax, indicate that two independent apoptotic pathways are activated in rods and cones in *Rpe65*-deficient LCA disease. This highlights the importance to decipher the specific death signaling pathways committed in rods and cones and understand their relative contribution to retinal defect toward the development of complementary and effective therapeutic treatments.

## Materials and Methods

### Mouse lines and genotyping

These studies adhered to the Association for Research in Vision and Ophthalmology (ARVO) statement for the use of animals in ophthalmic and vision research and were approved by the Veterinary service of the State of Valais (Switzerland). Wild-type C57BL/6 mice (wt) were purchased from Charles River Laboratories (Les Oncins, France). *Rpe65^−/−^* mice are on a C57BL/6 genetic background (from Dr T.M. Redmond, National Institutes of Health, Bethesda, USA) [4] and *Gnat1^−/−^* (from Dr. J. Lem, Tufts-New England Medical Center, Boston, USA) are on a BALB/c genetic background [54]. *Rpe65^−/−^/Gnat1^−/−^* double knockout mice were generated by crossbreeding *Rpe65^−/−^* mice with *Gnat1^−/−^* mice. Genotyping of the mice was determined by PCR analysis with genomic DNA isolated from tail tissue, as described [4], [54]. Animals were kept in a 12-h light/12-h dark cycle with unlimited access to food and water.

### Generation of *Rpe65^−/−^/Bax^−/−^* double knock-out mice

*Bax^−/−^* mice are homozygous for the Bax^tm1Sjk^ mutation (Charles River Laboratories). The targeted disruption of the *Bax* gene was performed in a 129-derived RW-G ES cell line and has been backcrossed 8 generations to C57BL/6. *Bax^−/−^* progeny have varying coat color, are poorly pigmented and have pink eye because the coat color loci tyrosinase (*Tyr*) and pink-eyed dilution (*p*) are linked to the Bcl2-associated X protein (*Bax*) gene. The strain is maintained through heterozygous matings since females are poor breeders and males are infertile. Heterozygous mice for *Bax* (+/−) and homozygous mice for *Rpe65* (−/−) were first crossbred to obtain *Bax^+/−^/Rpe65^+/−^* double heterozygous F1 animals. Double heterozygous F1 males and females were mated to generate *Bax^+/−^/Rpe65^−/−^* F2 mice both heterozygous for Bax (+/−) and homozygous for Rpe65 (−/−). F2 mice were then mated to obtain *Bax^−/−^/Rpe65^−/−^* double knock-out F3 animals used in this study. Bax-specific genotyping was performed by PCR analysis with genomic DNA isolated from tail tissue, as recommended by The Jackson Laboratory (http:// jaxmice.jax.org).

### Tissue isolation and RNA preparation

Age-matched animals were killed by cervical dislocation. Retinas from each mouse strain were dissected under a microscope to exclude extra-retinal tissues, and were quickly isolated in RNA*later* (Ambion, Huntingdon, United Kingdom) before being transferred in TRIzol (Invitrogen, Basel, Switzerland) and stored at −80°C until RNA extraction. Total RNA was extracted according to manufacturer's instructions and the amount of total RNA was determined by Ribogreen assay (Invitrogen).

### RT-PCR analysis

One µg of total RNA in a 20-µl reaction was used for cDNA synthesis using oligo (dT)_18_ according to the manufacturer's procedure (AffinityScript™ Reverse Transcriptase; Agilent, Basel, Switzerland). The equivalent of 50 ng original total RNA was used for PCR using the 2× Master Mix (Qiagen, Basel, Switzerland) and 1 µM forward and reverse primer pairs. PCR was performed with the following cycling conditions: 35 cycles of denaturation at 95°C for 30 sec, annealing at 55°C for 30 sec, and extension at 72°C for 30 sec. The primers used for detection of mouse *Bax* transcript were the following: forward primer 5′-CCAGGATGCGTCCACCAAGA-3′ and reverse primer 5′-GGTGAGGACTCCAGCCACAA-3′ (Eurogentec, Seraing, Belgium).

### Real-time PCR analysis

The equivalent of 50 ng original total RNA was used for PCR amplification using the 2× brilliant SYBR Green QPCR Master Mix (Agilent) with either 125 nM (*Gapdh*, *Gnat1*, *Gnat2*, *Crx*), 250 nM (*Bax*, *Swl opsin*, *Mwl opsin*, *Rhodopsin*, *Rom-1*, *Rds*) or 500 nM (*Bcl-2*) forward and reverse primer pairs, designed to span an intron of the target gene. Real-time PCR was performed in triplicate in a Mx3000PTM system (Agilent) with the following cycling conditions: 40 cycles of denaturation at 95°C for 30 sec, annealing either at 55°C (all genes except *Bcl-2*) or 60°C (*Bcl-2*) for 30 sec, and extension at 72°C for 30 sec. Quantitative values were obtained by the cycle number (Ct value) reflecting the point at which fluorescence starts to increase above background at a fixed threshold level. Values obtained for the target genes were normalized with the housekeeping gene *Gapdh*. Gapdh-normalized values for *Bax^−/−^* and *Rpe65^−/−^/Bax^−/−^* retinal RNA were additionally normalized with the photoreceptor marker gene *Crx* to compensate for the increased thickness of the whole retina due to increased cell number present in the INL and GCL of these mouse strains. For primer sequences, see Table S1.

### Histological analysis

Eyes were fixed in 4% paraformaldehyde (PFA)/phosphate-buffered saline (PBS) for 45 min, followed by cryoprotection in 30% sucrose/PBS. Ten µm-embedded frozen sections were stained with hematoxylin and eosine for light microscopy histology examination. For each individual eye (n = 4), four retina sections of various depths were stained for histology. For each retina section, a count of rows of photoreceptors in the ONL was performed from two different areas in the central retina on each side of the optic nerve, across both the superior and inferior hemispheres. In each area, five adjacent rows of nuclei from three individual fields were counted and the resulting numbers from each individual retina were averaged.

### Immunohistochemistry and peanut agglutinin (PNA) staining

Eyes were fixed in 4% PFA/PBS for 45 min, followed by cryoprotection in 30% sucrose/PBS. Ten µm-embedded frozen sections were further processed for immunohistochemistry. Briefly, frozen retina sections were blocked in PBS with 2% normal goat serum (Sigma, Buchs, Switzerland) and 0.2% Triton X-100 (Sigma) for 1 h at room temperature (RT) and incubated with primary antibodies in the blocking buffer overnight at 4°C. Sections were blocked again in blocking buffer for 30 min at RT before to be incubated with fluorochrome-conjugated secondary antibody for 1 h at RT. Incubation with secondary antibody alone was used as a negative control. Species and dilutions of the antibodies used were as follows: rabbit anti-gnat1 (1∶1'000; Calbiochem, San Diego, USA), rabbit anti-gnat2 (1∶500; Santa Cruz Biotechnology, Santa Cruz, USA) and Alexa Fluor 594 goat anti-rabbit IgG (1∶1'000; Invitrogen) or Alexa Fluor 488 goat anti-rabbit IgG (1∶500; Invitrogen). For PNA staining, retina sections were incubated at RT with fluorescein-congugated PNA (Sigma) used at 20 µg/ml for 75 min. Following 3 washes in PBS, sections were mounted in Citifluor AF1 (Citifluor Ltd, London, United Kingdom). Tissue sections were counterstained with 4′,6-diamidino-2-phenylindole, dihydrochloride (DAPI; Invitrogen) to identify retinal cell layers.

### Terminal dUTP Nick End-Labeling (TUNEL) of fragmented DNA

DNA strand breaks in retinal cell nuclei were detected by TUNEL assay on retina flatmounts. Enucleated eyes were fixed in 4% PFA/PBS for 5 min at RT, followed by dissection of the retina in PBS, after removal of cornea and lens, and additional fixation of the retina with photoreceptors faced up in 4% PFA/PBS for 60 min at RT. Each retina was then transferred on (3-Aminopropyl)triethoxysilane (APTES; Sigma)-treated slide and flattened into 4 quadrants by making incisions each 90° apart, from the ora serrata and stopping short of the optic nerve opening. Retina flatmounts on microscope slides were stored at −20°C until used. Before TUNEL staining, retinas were rehydrated in PBS for a few seconds and fixed in 4% PFA/PBS for 10 min at RT. The tissue was then dehydrated 2 minutes in each graded alcohol (2 times 95%, 2 times 100%), and defatted in xylene overnight to allow for better penetration across the outer limiting membrane of the retina. The following day, retina flatmounts were rehydrated in alcohol (2 times 100%, once 95%, once 80%) and in PBS, then permeabilized with 0.3% Triton X-100 for 15 min at RT, and finally digested with proteinase K (20 µg/ml; Roche, Rotkreuz, Switzerland) for 2 h at 37°C before TUNEL staining with terminal deoxynucleotidyl transferase (TdT) and TMR nucleotides (Roche) according to manufacturer's instructions. Retina flatmounts were further counterstained with DAPI (Invitrogen) to identify photoreceptor nuclei, followed by three washes in PBS, before to be mounted in Citifluor AF1 (Citifluor). For each retina flatmount, apoptotic cells were counted in at least three to four areas of each 4 quadrants and the resulting numbers from each retina flatmount (n = 3–5) were averaged.

### Imaging

Images were viewed under a fluorescence microscope equipped with a digital camera (Olympus BX61; Olympus, Lausanne, Switzerland) using appropriate filters.

### Statistical analysis

All results were expressed as means±SE of the indicated number of experiments. Statistical significance was calculated with the ANOVA test followed by Bonferroni post test adjustment.

## Supporting Information

Table S1 Supplemental table S1. Nucleotide sequences of primers used in real-time PCR.
(0.04 MB PDF)

Figure S1 Disruption of Bax in Rpe65-deficient Mice (A) PCR screening from genomic DNA showed specific amplification of the corresponding wild-type (wt) and mutant (mut) alleles from wt, Rpe65−/−, Bax−/− and Rpe65−/−/Bax−/− mouse genotypes. Genomic DNA from heterozygous Bax mice (Bax+/−) was used as control of PCR amplification of both alleles in a single reaction. (B) RT-PCR analysis confirmed disruption of Bax transcript in Bax−/− and Rpe65−/−/Bax−/− retinas, while Bax-specific RT-PCR product of the expected size (394-bp amplicon spanning exons 3 to 6) was observed in wt and Rpe65−/− retinas. Gapdh transcript amplification (287-bp spanning exons 3 to 5) was perfomed as control. Sample without cDNA template (dH2O) was used as a control of PCR specificity. MW, DNA ladder in base pairs.
(3.33 MB TIF)

Figure S2 Downregulated Expression of Anti-apoptotic Bcl-2 Was Not Restored in Rpe65-deficient Mice Lacking Bax Quantitative PCR analysis of Bcl-2 mRNA expression in 6 month-old mice showing that decreased expression in Rpe65−/− retinas was not restored in Rpe65−/−/Bax−/− retinas, as compared with wt retinas. Data are the mean±SE of three independent experiments. * p<0.001 by ANOVA test for Rpe65−/− and Rpe65−/−/Bax−/− versus wt.
(0.79 MB TIF)
