# Supplementary material for: Bax-Induced Apoptosis in Leber's Congenital Amaurosis: A Dual Role in Rod and Cone Degeneration
Source: PLoS One. 2009 Aug 12;4(8):e6616. doi: 10.1371/journal.pone.0006616 (PMC2720534; doi:10.1371/journal.pone.0006616)
Supplement: Table S1 — Supplemental table S1. Nucleotide sequences of primers used in real-time PCR. (0.04 MB PDF) [file pone.0006616.s001.pdf]

Gene	Strand	Nucleotide sequence (5’ - 3’)
Gnat1	Forward	AGA GGA TGC TGA GAA GGA TG
	Reverse	ACT GAA TCT TGA GCG TGG TC
Rhodopsin	Forward	CTC CAT GCT GGC AGC GTA CA
	Reverse	TGC TCA TCG GCT TGC AGA CC
Rom-1	Forward	CCA TGA AGT GCT GCT GGA AC
	Reverse	GGC CTC AGC TAG AAC TTC CT
Rds	Forward	GGC TTA CGG ACT CAA GAA TG
	Reverse	GTG AGC TGG TAC TGG ATA CA
Gnat2	Forward	CAT CAG TGC TGA GGA CAA AG
	Reverse	GAC TGG AAC TCT AGG CAC TC
SWL opsin	Forward	TGG TCA ACA ATC GGA ACC AC
	Reverse	AGG GCC AAC TTT GCT AGA AG
MWL opsin	Forward	TGA GGA TAG CAC CCA TGC AA
	Reverse	GGC GCA GCT TCT TGA ATC TC
Bax	Forward	GGC TGG ACA CTG GAC TTC CT
	Reverse	GGT GAG GAC TCC AGC CAC AA
Bcl-2	Forward	TTC GCA GAG ATG TCC AGT CA
	Reverse	TTC AGA GAC AGC CAG GAG AA
Crx	Forward	GCT TCT GCT TTC TGT TCT TC
	Reverse	AAC TTC CAG GCA CTC TGA TC
Gapdh	Forward	GAG GCC GGT GCT GAG TAT GT
	Reverse	GGT GGC AGT GAT GGC ATG GA
