# Supplementary figures and images for: Bax-Induced Apoptosis in Leber's Congenital Amaurosis: A Dual Role in Rod and Cone Degeneration
Source: PLoS One. 2009 Aug 12;4(8):e6616. doi: 10.1371/journal.pone.0006616 (PMC2720534; doi:10.1371/journal.pone.0006616)

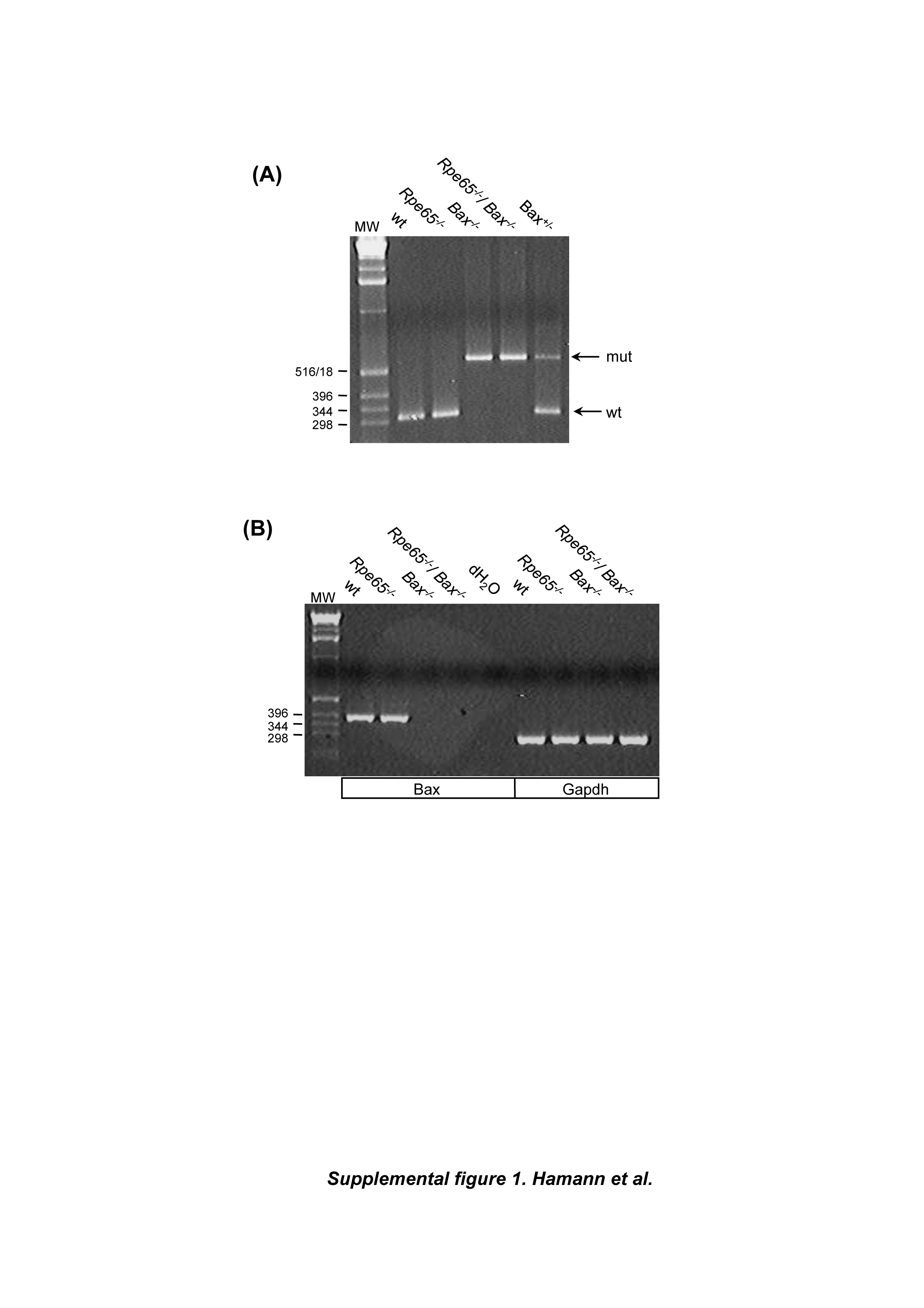

Supplement: Figure S1 — Disruption of Bax in Rpe65-deficient Mice (A) PCR screening from genomic DNA showed specific amplification of the corresponding wild-type (wt) and mutant (mut) alleles from wt, Rpe65−/−, Bax−/− and Rpe65−/−/Bax−/− mouse genotypes. Genomic DNA from heterozygous Bax mice (Bax+/−) was used as control of PCR amplification of both alleles in a single reaction. (B) RT-PCR analysis confirmed disruption of Bax transcript in Bax−/− and Rpe65−/−/Bax−/− retinas, while Bax-specific RT-PCR product of the expected size (394-bp amplicon spanning exons 3 to 6) was observed in wt and Rpe65−/− retinas. Gapdh transcript amplification (287-bp spanning exons 3 to 5) was perfomed as control. Sample without cDNA template (dH2O) was used as a control of PCR specificity. MW, DNA ladder in base pairs. (3.33 MB TIF) [file pone.0006616.s002.tif]

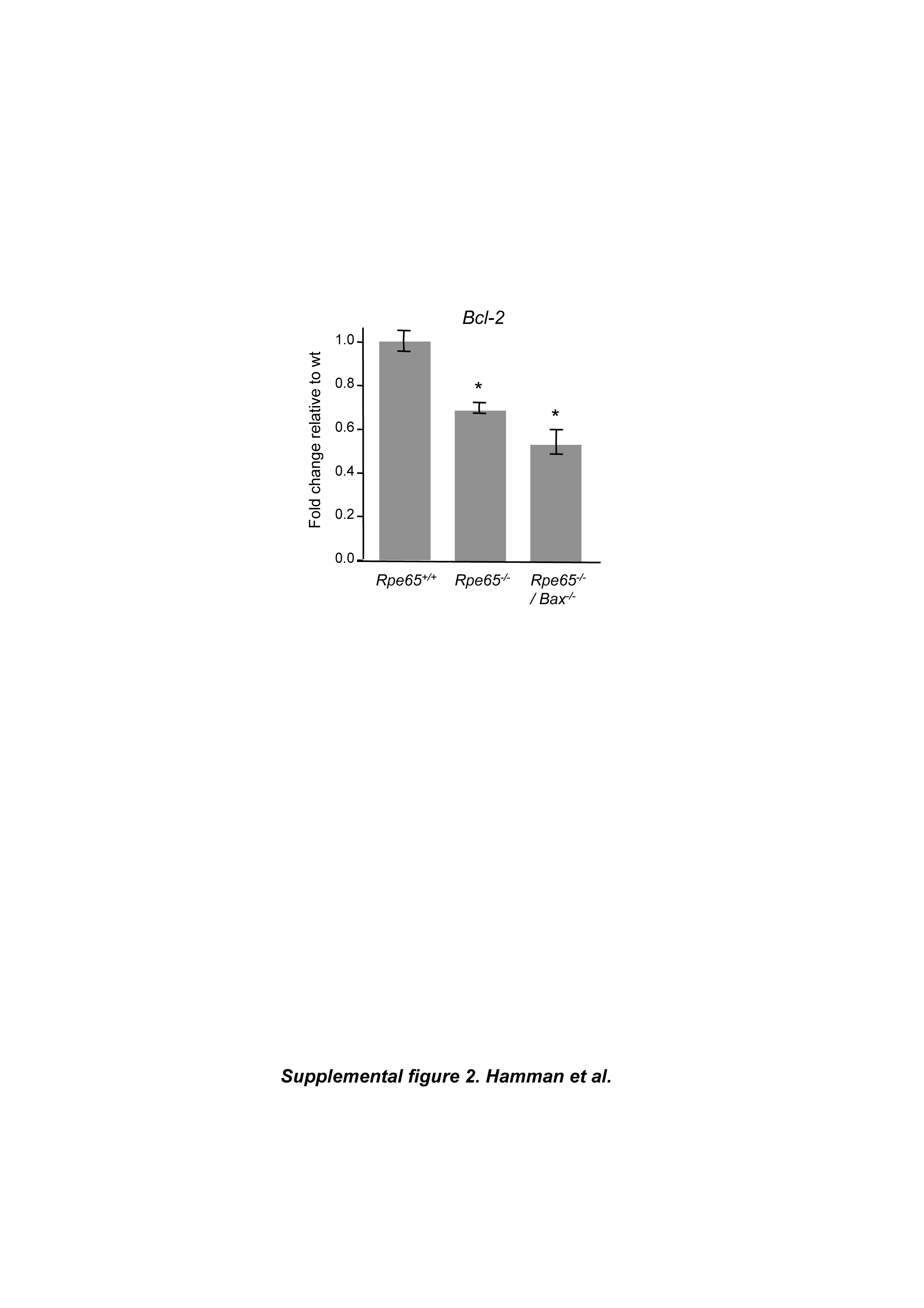

Supplement: Figure S2 — Downregulated Expression of Anti-apoptotic Bcl-2 Was Not Restored in Rpe65-deficient Mice Lacking Bax Quantitative PCR analysis of Bcl-2 mRNA expression in 6 month-old mice showing that decreased expression in Rpe65−/− retinas was not restored in Rpe65−/−/Bax−/− retinas, as compared with wt retinas. Data are the mean±SE of three independent experiments. * p<0.001 by ANOVA test for Rpe65−/− and Rpe65−/−/Bax−/− versus wt. (0.79 MB TIF) [file pone.0006616.s003.tif]
